# Is the role of probiotics friendly in the treatment of periodontal diseases !!

**DOI:** 10.4103/0972-124X.51892

**Published:** 2009

**Authors:** D Deepa, D. S. Mehta

**Affiliations:** 1 *Reader, Department of Periodontics, Subharti Dental College and Hospital, Meerut, UP, India*; 2 *Professor and Head, Department of Periodontology and Implantology, Bapuji Dental College and Hospital, Davanagere, Karnataka, India*

**Keywords:** Bifidobacterium, lactobacillus, probiotics

## Abstract

Probiotics utilize naturally occurring bacteria to confer a health benefit when administered in adequate amounts. A few conventional foods containing probiotics are yogurt, fermented and unfermented milk, soy beverages etc. Most often, they come from two groups of bacteria, *Lactobacillus or Bifidobacterium*. Probiotics have been extensively studied for their health promoting effects. Scientific understanding of probiotics and their potential for preventing and treating periodontal conditions is at its infancy, but moving ahead. Extensive research to create a probiotic product intended to maintain dental and periodontal health is needed.

## INTRODUCTION

Probiotics are live microorganisms administered in adequate amounts with beneficial health effects on the host. Not all bacteria are bad. In fact, beneficial microbes could represent the future of medicine. Antibiotics destroy the harmful bacteria that can cause infection, while also destroying the good bacteria that help to fight infection. Probiotics, on the other hand, re-populate the beneficial bacteria which can help kill pathogenic bacteria and fight against infection. Oral administration of probiotics may also benefit oral health by preventing the growth of harmful microbiota or by modulating mucosal immunity in the oral cavity.[1]

The application of selected beneficial bacteria, as an adjunct to scaling and root planing, would also inhibit the periodonto-pathogen recolonization of periodontal pockets and thus achieve and maintain periodontal health.[2] With the number of bacteria-resistant diseases on the rise and the length of time it takes to develop new antibiotics, it might be time to consider another alternative, “*Probiotics*” in the treatment of periodontal disease.

Lilley and Stillwell were the first to use the term “Probiotics”. Parker defined probiotics as organisms and substances which contribute to intestinal microbial balance. Fuller redefined probiotics as “A live microbial feed supplement which beneficially affects the host animal by improving its intestinal microbial balance”. Probiotics can help prevent and treat disease through several mechanisms.

1. Direct interaction: Probiotics interact directly with the disease-causing microbes, making it harder for them to cause the disease.
2. Competitive exclusion: Beneficial microbes directly compete with the disease, developing microbes for nutrition or enterocyte adhesion sites.
3. Modulation of host immune response: Probiotics interact with and strengthen the immune system and help prevent disease.

## PROBIOTICS AND PERIODONTAL HEALTH

Periodontitis is a multifactorial disease that encompasses the hard and soft tissue, microbial colonization (with or without invasion), inflammatory responses and adaptive immune responses. The complexity of the local tissue components, including bacteria and/or their products and virtually all aspects of host response mechanisms, has complicated our ability to elucidate the critical protective functions in the tissues and has continually provided evidence for the potential of host destructive factors as the ultimate causative parameters in the disease.[3] Treatment of periodontal diseases in recent years has moved towards an antibiotic/antimicrobial model of disease management. Probiotics might be a promising area of research in the treatment of periodontitis.

Probiotics lower the pH so that plaque bacteria cannot form dental plaque and calculus that causes the periodontal disease. They make an excellent maintenance product because they produce antioxidants. Antioxidants prevent plaque formation by neutralizing the free electrons that are needed for the mineral formation. Probiotics are able to breakdown putrescence odours by fixating on the toxic gases (volatile sulphur compounds) and changing them to gases needed for metabolism.

The most common probiotic strains belong to the genera *Lactobacillus* and *Bifidobacterium*. However, the *lactobacillus* species from which probiotic strains have been isolated include *L. acidophilus, L. johnsonii, L. casei, L. rhamnosus, L. gasseri, and L. reuteri.* Similarly, the *bifidobacterium strains include B. bifidum, B. longum, and B. infantis. Lactobacilli* can produce different antimicrobial components including organic acids, hydrogen peroxide, low-molecular weight antimicrobial substances, bacteriocins and adhesion inhibitors and have gained prominence as probiotics.

*Streptococcus Oralis* and *Streptococcus Uberis* have been shown to inhibit the growth of pathogens both in the laboratory and animal models. Presence of *S. Oralis* and *S. Uberis* provided a good indication of health of periodontium. When these bacteria are absent from sites in the periodontal tissues, those sites are more prone to disease.[4] The probiotic tablets (Wakamate D^®^; Wakamoto Pharmaceutical Co., Tokyo, Japan) contained 6.7×10^8^ colony forming units (CFU)/tablet of *L. salivarius* WB21 and xylitol (280 mg/tablet) were originally prepared to contribute for the intestinal microbial balance by providing acid tolerant *L. salivarius* WB21. Using these tablets, it was found that orally administered *L. salivarius* WB21 significantly decreased the plaque index and probing pocket depth of subjects who were smokers, suggesting clinical improvement of the periodontal condition by probiotic intervention. A significant reduction in salivary lactoferrin (*Lf)* levels was also observed for smokers at eight weeks.[1]

Very recently, Koll *et al*,[5] characterized 22 strains of orally isolated *lactobacilli* with regard to antimicrobial activities on oral pathogens including periodontopathic bacteria and tolerance to environmental stress *in vitro*.

A majority of the strains including L. salivarius were shown to suppress the growth of *Aggregatibacter actinomycetemcomitans* (formerly *Actinobacillus actinomycetem-comitans*), *P. gingivalis* and *Prevotella intermedia.* Probiotic strains included in periodontal dressings at optimal concentration of 10^8^ CFU/ml were shown to diminish the number of most frequently isolated periodontal pathogens, *Bacteroides species, Actinomyces sp., S. intermedius* and *Candida albicans.*

Teughels *et al*,[2] reported that the subgingival application of a bacterial mixture including *Streptococcus sanguis, S. salivarius,* and *Streptococcus mitis* after scaling and root planing significantly suppressed the re-colonization of *Porphyromona gulae* (canine *P. gingivalis)* and *P. intermedia* in a beagle dog model. This guided pocket recolonization approach may provide a valuable addition or alternative to the armamentarium of treatment options for periodontitis.

Passive immunization of humans using *Porphyromonas gingivalis* monoclonal antibodies temporarily prevents colonization of *P. gingivalis.* Probiotic therapy may be an alternative approach, but regulatory and safety issues for human periodontal vaccine trials must be considered. It has been shown to have a definite inhibitory effect on the production of volatile sulphur compounds (VSC) by *F. nucleatum* after ingestion of *Weissella cibaria* both *in vitro* and *in vivo*. The possible mechanism in the VSC reduction is the hydrogen peroxide generated by *W.cibaria* that inhibits the proliferation of *F. nucleatum*.

## CONCLUSION

Probiotics play an important role in combating issues with overuse of antibiotics and antimicrobial resistance. Today's new technological era would be the right time to change the way bacteria are treated. Further studies to understand the ability of probiotic bacteria to survive, grow, and have a therapeutic effect when used for treatment or when added to foods, to fix the doses and schedules of administration of probiotics. Hence, systematic studies and randomized controlled trials are needed to find out the best probiotic strains and means of their administration in different oral health conditions. Finally, possibilities to genetically modify or engineer potential probiotic strains may offer all new visions. Better scientific understanding and extended research of these tiny forms of life and their effect on humans in the treatment of periodontal diseases might further broaden the field of potential applications.
